# A Single Nucleotide Polymorphism (rs3811792) Affecting Human *SCD5* Promoter Activity Is Associated with Diabetes Mellitus

**DOI:** 10.3390/genes13101784

**Published:** 2022-10-03

**Authors:** Veronika Zámbó, Gabriella Orosz, Luca Szabó, Kinga Tibori, Szabolcs Sipeki, Krisztina Molnár, Miklós Csala, Éva Kereszturi

**Affiliations:** Department of Molecular Biology, Semmelweis University, H-1085 Budapest, Hungary

**Keywords:** type 1 diabetes mellitus (T1DM), type 2 diabetes mellitus (T2DM), stearoyl-CoA desaturase-5 (SCD5), single nucleotide polymorphism, lipotoxicity, fatty acid, lipid metabolism, metabolic disorder

## Abstract

The combined prevalence of type 1 (T1DM) and type 2 (T2DM) diabetes mellitus is 10.5% worldwide and this is constantly increasing. The pathophysiology of the diseases include disturbances of the lipid metabolism, in which acyl-CoA desaturases play a central role as they synthesize unsaturated fatty acids, thereby providing protection against lipotoxicity. The stearoyl-CoA desaturase-5 (SCD5) isoform has received little scientific attention. We aimed to investigate the *SCD5* promoter and its polymorphisms in vitro, in silico and in a case-control study. The *SCD5* promoter region was determined by a luciferase reporter system in HepG2, HEK293T and SK-N-FI cells and it was proved to be cell type-specific, but it was insensitive to different fatty acids. The effect of the *SCD5* promoter polymorphisms rs6841081 and rs3811792 was tested in the transfected cells. The T allele of rs3811792 single nucleotide polymorphism (SNP) significantly reduced the activity of the *SCD5* promoter in vitro and modified several transcription factor binding sites in silico. A statistically significant association of rs3811792 SNP with T1DM and T2DM was also found, thus supporting the medical relevance of this variation and the complexity of the molecular mechanisms in the development of metabolic disorders. In conclusion, the minor allele of rs3811792 polymorphism might contribute to the development of diabetes by influencing the *SCD5* promoter activity.

## 1. Introduction

About 537 million people worldwide have diabetes, the majority are living in high- and middle-income countries, and 1.5 million deaths are directly attributed to diabetes each year. Both the number of cases and the prevalence of diabetes have been steadily increasing over the past few decades [1,2]. Diabetes belongs to a group of metabolic disorders that are characterized by a long-term high blood glucose level due to either the inadequate production of insulin (type 1 diabetes mellitus, T1DM) or a poor response of the recipient cells to insulin (type 2 diabetes mellitus, T2DM). While the former is considered to be a chronic autoimmune disease [3], the latter is a condition that develops on the basis of obesity and a sedentary lifestyle [4]. However, in either type, the role of genetic determination in addition to environmental factors is established beyond doubt [5,6]. However, despite some overlap, the genetic factors of T1DM and T2DM currently appear to be distinct [7]. The accelerator hypothesis argues that T1DM and T2DM are the same disorder of insulin resistance, which are set against different genetic backgrounds. The hypothesis does not deny the role of autoimmunity, only its primacy in the process. It assumes that obesity, the excessive nutrient intake, and an imbalance of lipid metabolism are the main accelerating factors for the appearance of diabetic symptoms [8].

The balance of lipid metabolism in our body depends on a functionally appropriate combination of saturated (SFA), monounsaturated (MUFA) and polyunsaturated (PUFA) fatty acids (FAs) with different carbon chain lengths. Accordingly, changes in the FA distribution and composition have a significant impact on lipid metabolism and homeostasis, energy storage and many other lipid-related processes [9]. In mammalian cells, SFAs and MUFAs are the most abundant FAs, accounting for 80% of total FA content. The MUFA-producing human stearoyl-CoA desaturases (SCDs) are endoplasmic reticulum membrane-bound enzymes that introduce the first double bond in the *cis*-*Δ*9 position of a saturated fatty acyl-CoA, which are mainly palmitoyl-CoA and stearoyl-CoA that produce palmitoleyl-CoA and oleyl-CoA, respectively [10]. Two isoforms of SCDs have been identified in humans. Numerous studies have demonstrated the inevitable importance of stearoyl-CoA desaturase-1 (SCD1) in the metabolic and signaling pathways, making its role unquestionable in several prevalent human diseases, including obesity, diabetes, fatty liver, and cardiovascular diseases [11,12,13,14,15]. Much is known about the lipid-sensitive expression [16,17,18,19,20,21], rapid protein degradation [22,23], and complex transcriptional regulation [24] of SCD1.

Unlike SCD1, the second human SCD isoform, stearoyl-CoA desaturase-5 (SCD5), has received considerably less attention. In contrast to SCD1, which is expressed in the tissues of major importance in lipid metabolism (such as liver and adipose tissue) [25], SCD5 is more abundant in the brain, pancreas, and gonads [26,27]. Although the actual promoter region of the *SCD5* gene is largely unidentified, several transcription factor (TF) binding sites in the putative 5′ regulatory region have been predicted in silico that have previously been proved to regulate *SCD1* [16,28]. The multilevel FA-sensitive regulation, which has already been revealed for SCD1 [24,29], is still an open question in the case of SCD5 [30,31]. Despite the numerous studies focusing on the role of SCD5 in the regulation of lipid metabolism [32,33,34,35], the importance of this gene product in the mechanism of human metabolic diseases such as diabetes and obesity has been poorly characterized. Although SCD5 appears to be a major regulator of visceral fat deposition and distribution in a zebrafish model system [36], its polymorphisms have not been investigated at all in the research on the genetic basis of obesity-related conditions. Accordingly, our primary aims were to map the functional upstream regulatory region of the *SCD5* gene, to test the possible FA-sensitivity of its transcription, and to examine the SNPs in the region in silico, in vitro, and in an association analysis involving T1DM and T2DM patients.

In the present study, we identified the functional promoter region of the *SCD5* gene and examined the functional impact of rs6841081 and rs3811792 *SCD5* promoter polymorphisms and their potential association with diabetes. The *SCD5* gene expression was found to be insensitive to FAs, and it showed a pronounced tissue-specificity with a much higher promoter activity in the neural cells. The presence of the minor variant of rs3811792 SNP significantly reduced the activity of the *SCD5* promoter in vitro and modified the binding probability of several TFs in silico. A statistically significant association of rs3811792 SNP with both T1DM and T2DM was also found, thus supporting the medical relevance of this polymorphism and the complexity of the molecular mechanisms in the development of metabolic disorders.

## 2. Materials and Methods

### 2.1. Chemicals and Reagents

Culture medium and supplements were purchased from Thermo Fisher Scientific (Waltham, MA, USA). Oleate, palmitate, stearate, linoleate, elaidate, vaccenate, bovine serum albumin, HepG2, HEK293T, and SK-N-FI cells were purchased from Sigma-Aldrich (St. Louis, MO, USA). All of the chemicals that were used in this study were of analytical grade. All of the experiments and measurements were carried out by using Millipore ultrapure water.

### 2.2. Web-Based Tools for in Silico Transcription Factor Binding Analysis

The JASPAR (http://jaspar.genereg.net/, accessed on 30 June 2022) [37] open-access, non-redundant TF biding profile database was used to predict potential TF binding to the *SCD5* promoter that was affected by rs6841081 or rs3811792 polymorphism. Two other online available prediction programs (ALGGN-PROMO, http://alggen.lsi.upc.es/cgi-bin/promo_v3/promo/promoinit.cgi?dirDB=TF_8.3 [38], accessed on 18 July 2022 and LASAGNA-Search 2.0, https://biogrid-lasagna.engr.uconn.edu/lasagna_search/index.php [39]), accessed on 20 July 2022 were also used to confirm TF binding hits.

### 2.3. Reporter Plasmid Construction and Mutagenesis

Different sized fragments of *SCD5* upstream regulatory region were amplified using iProof™ High-Fidelity DNA Polymerase (Bio-Rad, Hercules, CA, USA) from human genomic DNA template using primers that contain *Kpn* I and *Hin*d III restriction endonuclease recognition sites. After purification and restriction endonuclease digestion (Thermo Fisher Scientific, Waltham, MA, USA), the amplicons were ligated (T4 Ligase, Thermo Fisher Scientific, Waltham, MA, USA) into pGL3B vector (Promega, Madison, WI, USA) that was upstream of the luciferase reporter gene. The studied natural variants were generated using Q5^®^ Site-Directed Mutagenesis Kit (New England BioLabs, Ipswich, MA, USA) following the manufacturer’s instruction. Mutagenic primers were designed using the online NEB primer design software, NEBaseChanger™. After digesting the original nonmutated and methylated plasmid by KLD reaction, an aliquot of constructs was transformed into XL10-Gold^®^ Ultracompetent Cells (Agilent, Santa Clara, CA, USA), which were then screened for positive colonies by single cell PCR. The cloning and mutagenic primers are listed in Table S1. All of the constructs were verified by Sanger sequencing them.

### 2.4. Cell Culture and Transfection

Human embryonic kidney (HEK293T), hepatocellular carcinoma (HepG2) and neuroblastoma (SK-N-FI) cells were cultured in 12-well plates (5 × 10^5^ cells per well) in Dulbecco’s modified Eagle medium (DMEM) which was supplemented with 10% fetal bovine serum and 1% penicillin/streptomycin solution at 37 °C in a humidified atmosphere containing 5% CO_2_. HEK293T cells were transfected with 0.5 μg pGL3B-SCD5_P1-P4 promoter constructs using 3 μL Lipofectamine 2000 (Invitrogen, Carlsbad, CA, USA) in 1 mL DMEM. HepG2 and SK-N-FI cells were transfected with 1 μg pGL3B-SCD5_P1-P4 promoter constructs using 3 μL Lipofectamine 3000 that was supplemented with 2 µL P3000 (Invitrogen, Carlsbad, CA, USA) in 1 mL DMEM. As a transfection control, 0.5 µg pCMV-*β*-gal plasmid was cotransfected into each sample. Cells were harvested and processed 24-30 h after transfection.

### 2.5. Cell Treatment

Oleate, palmitate, stearate, linoleate, elaidate, and vaccenate were diluted in ethanol (Molar Chemicals, Halásztelek, Hungary) to a final concentration of 50 mM and conjugated with 20% FA free BSA in 1:4 ratio at 50 °C for 1 h. The working solution for FA treatments was prepared freshly in FBS-free and antibiotic-free medium at 100 µM final concentration. The culture medium was replaced 5 h after transfection. The FA treatment was carried out for 24 h in 12-well plates.

### 2.6. Preparation of Cell Lysates

Cells were washed twice with PBS and harvested in 100 µL Reporter lysis buffer (Promega, Madison, WI, USA) was scraping and briefly vortexed. A single freeze–thaw cycle was followed by centrifuging in a benchtop centrifuge (5 min, max speed, 4 °C). Supernatants were used for enzyme activity determination.

### 2.7. Luciferase Assay

Luciferase activity was detected using the Luciferase Assay System kit (Promega, Madison, WI, USA) by adding 15 μL Luciferin reagent to 5 µL of all of the cell extracts. *β*-galactosidase activity of 20 µL cell lysates was measured by using *o*-nitrophenyl-*β*-D-galactopyranoside (at a final concentration of 3 mM) cleavage rate. Luminescence was detected using a Varioskan multi-well plate reader (Thermo Fisher Scientific, Waltham, Massachusetts, USA). Values for luciferase activity were normalized to *β*-galactosidase activity (measured by standard protocol using the same Varioskan plate reader in photometry mode). Each experiment was repeated three times independently, and each sample was studied in triplicate.

### 2.8. qPCR Analysis

Total RNA was purified from HepG2, HEK293T and SK-N-FI cells by using RNeasy Plus Mini Kit (Qiagen, Hilden, Germany) following the manufacturer’s instruction. Concentrations were measured using NanoDrop1000 spectrophotometer. To assess the integrity and purity of the isolated total mRNA samples, the ratios of their absorbance at 260/280 and 260/220 nm were determined, and they were also analyzed by agarose gel electrophoresis to visualize bands corresponding to 28S and 18S rRNAs, respectively. Human tissue RNAs were purchased from Thermo Fisher Scientific (Waltham, Massachusetts, USA). cDNA samples were produced by reverse transcription of 0.5 µg DNA-free RNA using SuperScript III First-Strand Synthesis System for RT-PCR Kit (Thermo Fisher Scientific, Waltham, Massachusetts, USA). Quantitative qPCR assay was performed in 20 µL final volume containing 5 µL 20× diluted cDNA, 1× PowerUp^TM^ SYBR^TM^ Green Master Mix, and 0.5 µM forward and reverse primers using QuantStudio 12K Flex Real-Time PCR System (Thermo Fisher Scientific, Waltham, Massachusetts, USA). *SCD5* sequences were amplified by 5′ ATG GAA ACC GGC CCT ATG AC 3′ and 5′ CCC CAG CCA GCA CAT GAA AT 3′ primer pairs. *GAPDH* cDNA was also amplified as an endogenous control using 5′ GTC CAC TGG CGT CTT CAC CA 3′ and 5′ GTG GCA GTG ATG GCA TGG AC 3′ primers. The first step of the thermocycle was an initial denaturation and enzyme activation at 95 °C for 2 min. It was followed by 40 cycles of 95 °C for 15 s, 55 °C for 15 s, and 72 °C for 1 min; measurement of the fluorescent signal was carried out during annealing. Reactions were performed in triplicates, and a reaction mixture with RNase-free water instead of template cDNA was employed as non-template control. Relative expression levels were calculated as 2^−Δ*C*^^T^, where Δ*C*_T_ values corresponded to the difference of the *C*_T_-values of the endogenous control and target genes.

### 2.9. Subjects

One hundred and forty-five patients that were diagnosed with type 1 diabetes mellitus (49.7% female, 50.3% male, disease onset at the age of 35.5 ± 13.1 years, 1-*β* for rs6841081: 0.0690, 1-*β* for rs3811792: 0.2866) and 253 patients that were diagnosed with type 2 diabetes mellitus (51.8% female, 48.2% male, disease onset at the age of 62.7 ± 12.1 years, 1-*β* for rs6841081: 0.0761, 1-*β* for rs3811792: 0.3749) in the 2^nd^ Department of Internal Medicine, Semmelweis University were recruited in the study. The control group consisted of 350 volunteers without medical history of any metabolic disease (61.0% female, 39.0% male, mean age: 31.1 ± 20.1 years). The diagnosis of diabetes was made based on fasting blood sugar values, oral glucose tolerance test (OGTT), and HbA_1C_ value according to WHO regulations. Individuals with autoimmune, infectious, or metabolic disorders other than type 1 or 2 diabetes were excluded from the study. Genetic analysis of the participants was approved by the Local Ethical Committee (ETT-TUKEB ad.328/KO/2005, ad.323-86/2005-1018EKU from the Scientific and Research Ethics Committee of the Medical Research Council). Participants signed written informed consent documents before sample collection was performed for genetic analysis to take place. In order to avoid the risk of spurious association due to population stratification, only subjects of Hungarian origin were included to ensure the comparison of homogenous populations. Buccal epithelial cells were collected using swabs. The first step of the DNA isolation was the incubation of the buccal samples at 56 °C overnight in 0.2 mg/mL Proteinase K cell lysis buffer. Subsequently, proteins were denatured using saturated NaCl solution. DNA was then precipitated using isopropanol and 70% ethanol. DNA pellet was resuspended in 100 µL 0.5× TE buffer (1× TE: 10 mM Tris pH = 8.0; 1 mM EDTA). Concentrations of the samples were measured using NanoDrop1000 spectrophotometer.

### 2.10. Genotyping

Rs6841081 and rs3811792 polymorphisms of the *SCD5* gene were genotyped using pre-designed TaqMan assays (C_34192814_10 and C_27029625_20, Thermo Fisher Scientific, Waltham, MA, USA). qPCR assay was performed in 5 µL final volume containing approximately 4 ng genomic DNA, 1× TaqPath™ ProAmp™ Master Mix, and 1× TaqMan^®^ SNP Genotyping Assay using QuantStudio 12K Flex Real-Time PCR System (Thermo Fisher Scientific, Waltham, MA, USA). Thermocycle was started by activating the hot start DNA-polymerase and denaturing genomic DNA at 95 °C for 10 min. This was followed by 40 cycles of denaturation at 95 °C for 15 sec, and combined annealing and extension at 60 °C for 1 min. Real-time detection was carried out during the latter step to verify the results of the subsequent post-PCR plate reads and automatic genotype calls.

### 2.11. Statistical Analysis

Relative luciferase activities and mRNA levels are presented in the diagrams as mean values ± S.D. and were compared by ANOVA with Tukey’s multiple comparison post hoc test using GraphPad Prism 6 software. Differences with a *p* < 0.05 value were considered to be statistically significant. Genotype–phenotype association was assessed by *χ*^2^-test comparing the genotype distribution of the patient and the control groups (i.e., additive model). Odds ratios were calculated by comparing the groups with and without the risk allele. The level of statistical significance was adjusted after Bonferroni correction. The statistical power for both patient groups and for both SNPs was calculated based on a likelihood ratio test framework using the additive model option [40].

## 3. Results

### 3.1. Cell Type-Specific Promoter Activity of Human Stearoyl-CoA Desaturase-5

**Figure 1:**
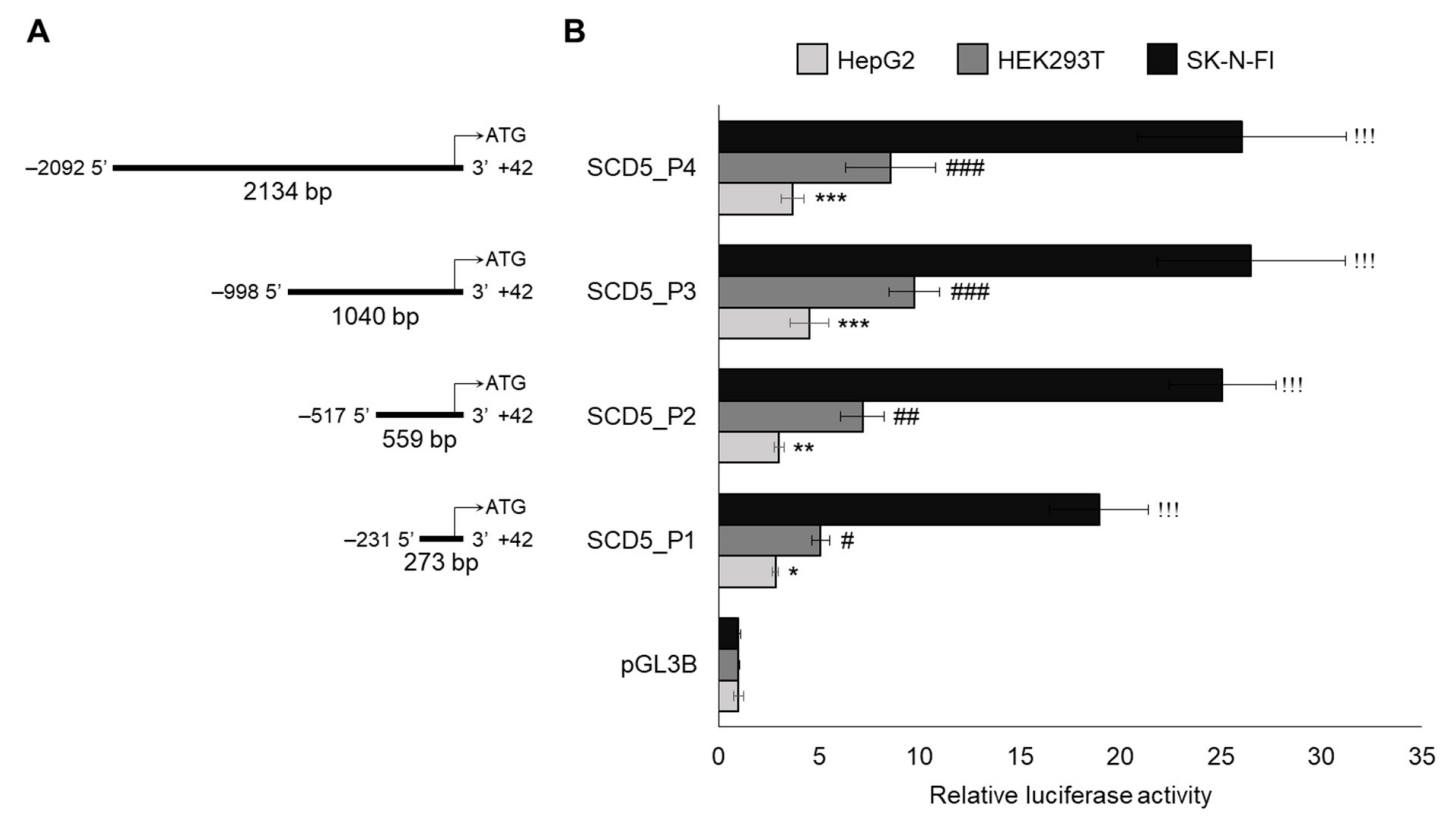
Relative promoter activity of different constructs of human *SCD5* gene promoter in three different human cell lines. The length and position of the subcloned *SCD5* 5′ regulatory sequences are numbered from the translation start site (+1) (**A**) and their relative luciferase activity that is measured in HepG2, HEK293T, and SK-N-FI cell lines is presented. (**B**) pCMV-*β*-gal vector served as transfection control. Luciferase and *β*-galactosidase enzyme activities were measured as indicated in Section 2, and their relative ratios are shown as bar graphs. The diagram depicts the results of three independent measurements that are normalized to pGL3B “empty” promoterless vector. Data are shown as mean values ± S.D. Statistical analysis was performed by using the Tukey–Kramer Multiple Comparisons Test. * or #: *p* < 0.05; ** or ##: *p* < 0.01; ***, !!!, ###: *p* < 0.001.

The first aim of our work was to identify the functional promoter of *SCD5*. Four increasingly larger sizes of DNA segments of the 5′ regulatory region were cloned into the pGL3B luciferase reporter vector, the exact lengths and positions of these are shown in Figure 1A.

The *SCD5* promoter constructs and the pCMV-*β*-gal vector were transiently transfected into HepG2, HEK293T and SK-N-FI cells, and the luciferase activity of the cell lysates was measured and normalized to *β*-galactosidase 24-30 h after the transfection. All four of the promoter constructs presented significantly higher relative luciferase activity than the promoterless pGL3B did, but the 1040 bp construct SCD5_P3 showed the highest activity in each cell line (Figure 1B). However, the increment in the relative promoter activity differed greatly among the cell lines. While the SCD5_P3 construct showed only a 4.5-fold increase in HepG2, it caused a nearly 10-fold elevation in HEK293T (Figure 1B) and an even more pronounced, approximately 26-fold increase in the SK-N-FI cells of neural origin (Figure 1B).

**Figure 2:**
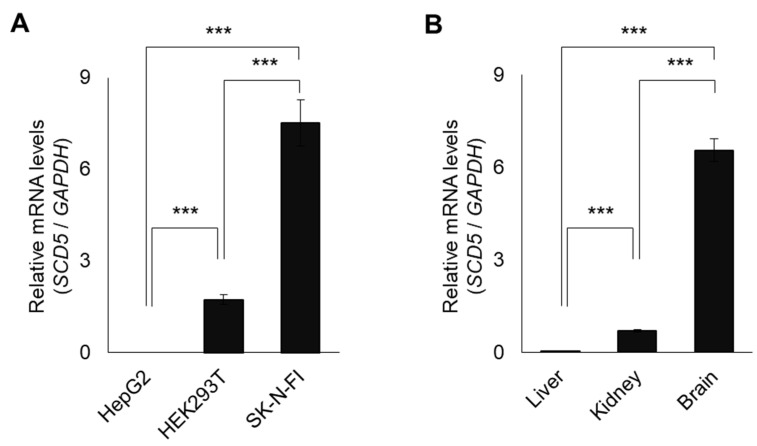
*SCD5* mRNA expression in three different cell lines (**A**) and human tissues. (**B**) The mRNA expression was measured in HepG2, HEK293T, and SK-N-FI cells, as well as in human liver, kidney, and brain samples. Samples were prepared as described in Section 2. qPCR was performed using *GAPDH* and *SCD5* sequence specific primers as indicated in Section 2. The diagram depicts the results of three independent measurements. Statistical analysis was performed by using the Tukey–Kramer Multiple Comparisons Test. Data are shown as mean values ± S.D. ***: *p* < 0.001.

An analysis of the endogenous *SCD5* expression in the three cell lines revealed that there were almost completely undetectable traces of *SCD5* mRNA in the liver-derived HepG2 cells, while the HEK293T and SK-N-FI cells showed moderate and high mRNA levels of it, respectively (Figure 2A).

A very similar pattern was observed when performing the comparison of the human tissue RNA samples (Figure 2B). In contrast to the minimal hepatic *SCD5* expression, the mRNA levels were one order of magnitude higher in the kidney tissue and two orders of magnitude higher in the brain tissue. These findings are in accordance with the results of our luciferase assays, which indicate that there is a cell type-specific promoter activity in the *SCD5* gene.

### 3.2. Fatty Acid Insensitive Promoter Activity of Stearoyl-CoA Desaturase-5

**Figure 3:**
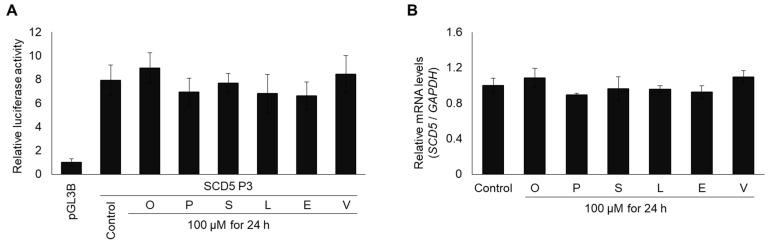
Effect of different fatty acids on relative *SCD5* promoter activity (**A**) and on mRNA level (**B**). (**A**) Transfection and FA treatment were performed as described in Section 2. pCMV-*β*-gal vector served as transfection control. Luciferase and *β*-galactosidase enzyme activities were measured as indicated in Section 2 and their relative ratios are shown as bar graphs. The diagram depicts the results of three independent measurements that were normalized to pGL3B “empty” promoterless vector. (**B**) The mRNA expression was measured in FA treated HEK293T cells. Samples were treated and prepared as described in Section 2. qPCR was performed using *GAPDH* and *SCD5* sequence specific primers as indicated in Section 2. The diagram presents the results of three independent measurements. Data are shown as mean values ± S.D. Statistical analysis was performed by using the Tukey–Kramer Multiple Comparisons Test.

As the expression of the *SCD1* gene has been shown to be FA sensitive [16,17,18,19,20,21], this possibility arose in the case of *SCD5* as well. We aimed to test this hypothesis by using six different FAs (oleate, palmitate, stearate, linoleate, elaidate, and vaccinate) in the luciferase assay, but to ensure the reliability of the model, we first tested the possible effect of these FAs on the luciferase activity using HEK293T cells that were transfected with the control pGL3B vector. A mild FA sensitivity was observed, and a significantly higher luciferase activity was detected while it was in the presence of stearate in comparison to that of the untreated sample (Figure S1); therefore, we decided to normalize the luciferase/*β*-galactosidase activity of each sample to that of the control pGL3B which was treated with the same FA. When the SCD5_P3 construct was tested for FA sensitivity in the optimized experimental system, none of the six FAs affected the relative luciferase activity when the promoter activity was compared to that of the untreated cells (Figure 3A).

Furthermore, these FAs had no effect on the endogenous *SCD5* mRNA levels either (Figure 3B). Collectively, these data imply that, unlike in case of *SCD1*, the expression of *SCD5* may be FA independent.

### 3.3. Effect of rs3811792 Polymorphism on Stearoyl-CoA Desaturase-5 Promoter Activity

**Figure 4:**
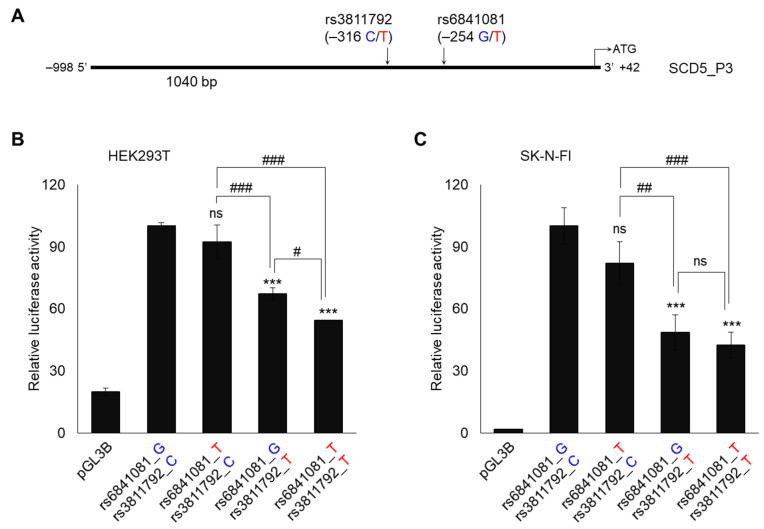
Position (**A**) and effect of two polymorphisms on relative *SCD5* promoter activity in HEK293T (**B**) and SK-N-FI (**C**) cells. (**A**) The position of two polymorphisms is marked on *SCD5* 5′ regulatory region and numbered from the translation start site (+1). Transfection was performed as described in Section 2. pCMV-*β*-gal vector served as transfection control. Luciferase and *β*-galactosidase enzyme activities were measured as indicated in Section 2 and their relative ratios are shown as bar graphs. The diagram depicts the results of three independent measurements normalized to the SCD5_P3 with the highest activity. Data are shown as mean values ± S.D. Statistical analysis was performed by using the Tukey–Kramer Multiple Comparisons Test. #: *p* < 0.05; ##: *p* < 0.01; *** or ###: *p* < 0.001; ns: not significant.

Based on the NCBI database, two SNPs (rs6841081, rs3811792, minor allele frequency > 1%) are located in the promoter of *SCD5*, and their position is depicted in Figure 4A.

All four of the possible combinations (i.e., haplotypes) of the two polymorphisms were created by a site-directed mutagenesis in the SCD5_P3 promoter construct, and their impact on the relative luciferase activity was examined in the HEK293T and SK-N-FI cells (Figure 4B,C). The plasmid carrying the higher frequency allele of both of the SNPs (rs6841081_G/rs3811792_C) resulted in the highest promoter activity in both of the cell lines. Although the presence of the minor allele of rs6841081 polymorphism (rs6841081_T/rs3811792_C) caused a mild reduction in the luciferase enzyme activity which was compared to that of the frequent haplotype version (rs6841081_G/rs3811792_C), this was not significant in either the HEK293T or the SK-N-FI cells (Figure 4B,C). In contrast, the minor allele of rs3811792 (rs6841081_G/rs3811792_T) resulted in a significantly lower promoter activity when it was compared to that of the frequent haplotype construct (rs6841081_G/rs3811792_C), and the reduction was of 30% in the human embryonic kidney-derived cell line (Figure 4B), and it was more than 50% in the neuroblastoma cells (Figure 4C). The haplotype of both of the minor alleles (rs6841081_T/rs3811792_T) showed a minimal further reduction of the *SCD5* promoter activity when it was compared to that of rs6841081_G/rs3811792_T, but this was statistically significant only in the HEK293T cell line (Figure 4B). In summary, it was the rs3811792 promoter SNP that negatively influenced the *SCD5* promoter activity in both of the cell lines that we examined in vitro in the luciferase reporter system.

### 3.4. Effect of rs6841081 and rs3811792 Promoter Polymorphisms on Transcription Factor Binding Sites in Silico

The possible impact of the two SNPs on the TF binding sites in the promoter of the *SCD5* gene was analyzed in silico using the JASPAR transcription factor binding site prediction program. Specifically, we addressed the question of whether the exchange of the two nucleotides that were affected by the polymorphisms could cause a predictable change in the binding probability of any of the TFs in this region. During the analysis, two 41-nucleotide long DNA sections were compared for each SNP, in which either version of the polymorphic nucleotide was located at position 21.

In the first step, the matrix of all (949) of the TFs that were included in the JASPAR database was compared with the DNA sequences that are described above. In order to find all of the possible TF binding sites, the TF binding probability was set to the lowest value (1%) that was allowed by the software. More than 56,300 hits were obtained (Table 1) for both of the allelic variants in case of each polymorphism.

By definition, the vast majority of these were very low probability hits. In the second step, the hit list was narrowed to identify the most relevant TFs. The TFs that were retained were those whose binding probability showed at least 15% difference between the two variations of the given polymorphism. Thus, the list of TFs was reduced to 438 for rs6841081 SNP and to 372 for rs3811792 SNP (Table 1). However, some of these hits could have a low binding probability, although they were significantly different for the two alleles. In the third step, therefore, only the TF hits were kept when they had a relative score that was above 80% for at least one allele (Table 1). The three-step filtering identified six TFs for rs6841081 SNP (Table 1 and Table 2) and nine TFs for rs3811792 SNP (Table 1 and Table 3) that are predicted to bind to the promoter sequence with a substantially high (at least 80%) probability, and their binding probability is remarkably (by at least 15%) different for the two allelic versions of the polymorphism.

Table 2 lists the names and IDs of the TFs that were affected by rs6841081 SNP as well as their relative binding probabilities to the two alleles and their differences.

The binding probabilities of the SPI1, MEIS3, SOX18, and NFIA factors are increased by the presence of the minor polymorphic T allele (Table 2, a red color and a darker shade means that there is a larger probability difference), while TFE3 and TFAP2A prefer to bind to the major polymorphic sequence (Table 2, a green color and a darker shade means that there is a larger probability difference). The results that were obtained with rs3811792 SNP are summarized in Table 3.

Out of the nine TFs, four of them (SOX10, SOX2, MEIS2, RBPJ, shown in red) are predicted to bind more strongly to the minor alleles containing the T nucleotide, while in the case of five TFs (NFATC2, ZNF354C, NFATC3, MZF1, ETS1, marked in green), the same T allele reduces the likelihood of an interaction.

**Figure 5:**
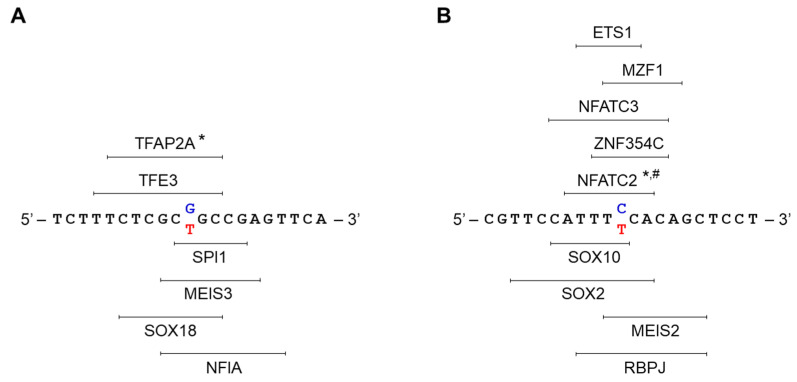
Transcription factor binding sites in the *SCD5* promoter that were affected by rs6841081 (**A**) and rs3811792 (**B**) polymorphisms. The major alleles of the SNPs are marked in blue, and the minor polymorphic versions are in red in the DNA sequences. The exact binding sites of the transcription factors that were identified by the JASPAR online prediction program and filtered as described in Section 3 are plotted. TFs that are typed above the sequence are more likely to bind to the major allele, while TFs that are below the sequence prefer to bind to the minor polymorphic allele. * indicates results confirmed by PROMO, # shows hits confirmed by LASAGNA.

The exact binding positions of the TFs that were affected by the two *SCD5* polymorphisms according to the in silico analyses are shown in Figure 5.

In the case of the rs6841081 SNP, TFAP2A was also confirmed to be present by the PROMO TF binding site search database, which is available online (Figure 5A). The NFATC2 binding site that was affected by the rs3811792 polymorphism was also predicted by two other programs (PROMO and LASAGNA) (Figure 5B).

### 3.5. Association of rs3811792 Polymorphism with T1DM and T2DM

The putative associations between the type 1 or type 2 diabetes mellitus and the rs6841081_G/T or rs3811792_C/T SNP were assessed by case–control setup. The observed genotype distributions of the control group for both of the polymorphisms were in accordance with the expected values that were calculated based on the Hardy–Weinberg equilibrium (*χ*^2^-test: *p* = 0.9769 for rs6841081, *p* = 0.9669 for rs3811792). The frequency of the minor rs6841081 SNP allele was 1.1% in the control population, which is in agreement with the European population average as per the NCBI database (0.9-2.8%). The 15.7% frequency of the minor rs3811792 SNP allele was slightly below the European population average (19%).

The genotype frequencies of the two SNPs that were measured in the healthy control population were compared with those that were found in the T1DM and T2DM groups using the *χ*^2^-test. Since this meant we had to conduct four different tests (two patient groups and two SNPs), the limit of the statistical significance was lowered from *p* < 0.05 to 0.0125 due to the Bonferroni correction. No association was found for the rs6841081 polymorphism either with T1DM or with T2DM (Table 4).

In contrast, the frequency of the rs3811792 SNP genotype was significantly different in both the T1DM (*p* = 0.0029, Table 4) and T2DM (*p* = 0.0114, Table 4) groups when they were compared to those of the control population. It is noteworthy that this difference can be largely attributed to the different frequency of the CT heterozygous genotype in the T2DM group (32% vs. 27%, Table 4) and to that of the TT homozygous genotype in the patients with T1DM (9% vs. 2%, Table 4). For this reason, different genotype categorizations were used for the determination of the odds ratios in the two patient groups. For T1DM, the patients with the C allele and the patients without the C allele were placed into separate categories, while in the case of T2DM, the T allele carriers and T allele non-carriers were grouped separately. In the absence of the C allele of rs3811792, the chance of developing T1DM was found to be increased by more than four times, although this is with the occurrence of a large confidence interval (Table 4). Despite there being significantly different genotype frequencies between the T2DM and control groups, the OR remained close to one for this SNP.

## 4. Discussion

Despite the progress that has been made in the research on SCD5 in recent years, many aspects of the transcriptional, nutritional, and hormonal regulation of this desaturase are still to be elucidated. To understand the exact function of SCD5 and its role in lipid metabolism, it would be necessary to clarify the regulation of its gene expression. The long overdue step toward the identification of the valid regulatory molecules that are involved in it is the mapping of the functional *SCD5* promoter region. In the work that is presented here, roughly 1000 base pair section in the 5′ regulatory region of *SCD5* were identified, which showed the highest activity in each cell line that we examined in vitro in a luciferase reporter system. These findings are in line with the preliminary expectations that we had since the average promoter length of human genes is between 300 and 1000 bp [41]. In addition, very similar results were obtained for *SCD1*, the other human desaturase isoform that was characterized previously in detail in a similar reporter system, with a sequence of nearly 1000 bp (between 882 and 150 nucleotides) that provides a maximum amount of transcriptional activity and was identified as the functional promoter [24]. Although several specific transcription factors influencing the *SCD1* gene expression have been already identified [24], and a lot is known about the hormonal and nutritional regulation of the *SCD1* gene [16], only in silico prediction data on *SCD5* are available. A number of TF binding sites at the 5′ regulatory region of human *SCD5* indicate that C/EBP-*α*, AP1, SP1, NF-1, NF-Y, T3R, PPAR-a, and SREBP1 may bind to the promoter sequence of the gene [28]. As the binding of the TFs to the aforementioned binding sites was known to control the expression of *SCD1* [16], and earlier studies assumed that there was both a similar function and a similar regulation for the two SCD isoforms, in silico searches have focused mainly on the similarities with SCD1.

However, them having a similar function seems less and less likely in light of the increasingly detailed mapping of the differences between the regulation of the two isoforms. The likelihood of the different transcriptional regulatory mechanisms of the two SCDs is also supported by the fact that the promoter activity of *SCD5* was not sensitive to the presence of different FAs in our experimental system. This observation is not without precedent, as the diets containing various unsaturated FAs, which have been shown to enhance the expression of *SCD1* in cows, left the *SCD5* mRNA level unchanged [30]. A similar phenomenon was also described in human tumor cell lines, where, in contrast to *SCD1*, neither the change in the serum lipid level [42] nor the presence of retinoic acid [35] affected the expression of the *SCD5* gene. The regulation of *SCD1*, but not *SCD5*, is affected significantly by the lipid-derived factors at different levels. It is known that the promoter activity of *SCD1* is influenced positively by the SFAs, and it is negatively influenced by the MUFAs and PUFAs [16,17,18,19,20,21,24]; in addition, oleate also enhances the intracellular degradation of the SCD1 protein [29,43]. Furthermore, a common missense polymorphism in the *SCD1* gene can stabilize the protein in an FA-dependent manner [23]. Although the two desaturases catalyze the same reaction, increasing evidence suggests that SCD5 may be less sensitive to the regulation by lipid factors in comparison to SCD1.

The different regulation also raises the possibility of it having a different function, which might be further supported by the significantly different tissue expression of the two isoforms, as well as the cell line-specific promoter activity of *SCD5*. It is well known that *SCD1* is ubiquitously found in all tissues, but it is more predominantly present in adipose tissue, liver tissue, brain tissue, heart tissue, breast tissue, and lung tissue [25], and while this is in agreement with our findings, *SCD5* is primarily expressed in the fetal and adult brain, the pancreas, the gonads, and to a lesser amount, in the kidney tissue, lung tissue, and adipose tissue [26,27]. Details of the evident tissue-specific transcriptional regulation are still unclear, and their discovery may shed light on the exact function and role of the enzyme in lipid metabolism and its related disorders.

Polymorphisms in the promoter region can modify gene expression. The present work is the first to functionally examine the two promoter SNPs of human *SCD5*, and it revealed that the T allele of rs3811792 reduces the *SCD5* promoter activity in the kidney-derived and neuronal cell lines. Interestingly, not only the SCD5, but also the human SCD1 polymorphisms have been quite neglected, and only a few variants have been characterized so far, and there is no promoter SNP among them, instead there is only the stabilizing effect of a missense variant [23] and the microRNA binding site-modifying role of a 3′ untranslated region (3′ UTR) polymorphism [44] have been described.

Just as certain polymorphisms can affect the microRNA binding sites (see above), other variations may largely modify the TF binding sites in the promoter. The in silico three-step analysis that we performed in the case of both polymorphisms identified that there are TFs whose binding probability is significantly affected by the exchange of the given nucleotide. Among these, NFATC2, which prefers the C allele of the rs3811792 polymorphic *SCD5* promoter, was confirmed by several predictive programs, and this polymorphism was also shown to modify the promoter activity and to be associated with diabetes. NFATC2 is a member of the nuclear factor of the activated T-cells family which plays a central role in inducing gene transcription during the immune response. Obesity and obesity-related conditions are associated with changes in the immune system that significantly hinder its ability to mount efficient immune responses [45]. Moreover, increased lipid loads can further dysregulate the defective T-cell responses, and based on our results, NFATC2, which is one of the potential mediators that is involved, may act in an allele-specific manner. In addition, an analysis of the 3′ UTR of the *SCD5* gene suggests the presence of several conserved regions for the microRNA families among vertebrates, including two sites for mammals [28]. Importantly, these microRNA clusters are associated with several diseases including non-alcoholic fatty liver disease, schizophrenia, autism, as well as brain and pancreatic cancers [28]. The T-cells from patients with rheumatoid arthritis are characterized by increased levels of microRNA 34b and a decrease in the level of *SCD5*, which is a target for this microRNA [31], thereby suggesting a further connection between the regulation of the desaturase levels and the immune-inflammatory functions in humans.

The present work is pioneering regarding not only the functional characterization of these factors, but also the association analysis of them, since no one has investigated the association of a single *SCD5* polymorphism with diabetes before, although it would be plausible given the function of SCD1. Based on our result, the genotype frequency of rs3811792 SNP significantly differs from the healthy population both in T1DM, which is primarily caused by autoimmune processes, and in T2DM, which is obesity-related and also characterized by immune response abnormalities and chronic inflammation. It is proved that SCD5 may contribute to the development of T2DM, since SCD5 was identified as a master regulator of fat distribution, which plays a significant role in determining the visceral adipose tissue accumulation, which is a major risk factor for diabetes [36]. On the other hand, the connection of SCD5 with T1DM may be also relevant due to the significantly higher expression of the enzyme in the pancreas in comparison to that in other tissues [26,27,46], even though a precise functional explanation is not yet available. At the same time, the association that we have demonstrated raises several additional questions. One such question that is yet to be answered is how the same allele of the same polymorphism can be related to both T1DM and T2DM. A possible explanation is offered by Wilkin’s accelerator hypothesis, according to which T1DM and T2DM are distinguished only by the time of the onset of the symptoms, with an earlier-onset diabetes reflecting a more susceptible genotype [8]. This may be reflected by the stronger association that is measured with the rs3811792 SNP in the T1DM population in comparison to that in the T2DM population (*p* = 0.0029 vs. *p* = 0.0114). It is also worth noting that in T1DM, the frequency of the TT genotype containing the minor allele increases significantly, while in T2DM, the frequency of CT heterozygotes differs from that of the control group. This can be further evidence for the spectrum nature of diabetes according to the accelerator hypothesis since it is conceivable that while the CT genotype leads to the late onset of the condition in association with other environmental factors, while the TT genotype manifests itself phenotypically at a younger age. In any case, it is certain that the two types of diabetes are complex multifactorial conditions that develop on the basis of several genetic and environmental factors, and so the rs3811792 SNP of the *SCD5* promoter may only be one factor in this complex system.

The study that is presented here aimed to shed light on SCD5, the so far neglected isoform of human stearoyl-CoA desaturases of potential pathological importance. Outlining the functional upstream promoter region was a necessary first step to investigate the transcriptional control of the gene expression. The maximal degree of promoter activity that was ensured by an approximately 1 kb segment was highly dependent on the cell type, and the pattern closely resembled the tissue specificity of in vivo *SCD5* expression. Although these results strongly indicate that the TFs that are responsible for the tissue specific activation or repression of *SCD5* bind to the upstream promoter, the contribution of possible downstream regulatory elements cannot be ruled out, and a thorough search for the relevant response elements should be extended accordingly. Since the study of the *SCD5* promoter and its polymorphisms was performed in a cellular system in vitro, it would be necessary to prove the effect of these human variations on transcription in vivo, as well as to confirm the in silico predicted allele-dependent TF binding by direct methods either in vitro or in vivo. Our findings also showed that *SCD5* expression is not sensitive to the different FAs, which is a remarkable difference in comparison to *SCD1* expression, and it is likely related to the somewhat different roles of the two isoforms at the cellular and particularly at the organismal level. Although apparently SCD1 and SCD5 catalyze the same biochemical reactions, the formation of unsaturated fatty acyl chains might have significantly diverse metabolic functions in various cells and tissues. Nevertheless, our in vitro findings do not pinpoint the exact nature of these differences, so this intriguing area requires further research. Due to the rather low number of cases that we used and in order to increase the statistical power of the study, the extension of the association analysis to larger control and patient groups should also be considered. Nevertheless, the association that we found between a common *SCD5* promoter polymorphism and diabetes mellitus not only extends the list of diabetes-related genes with another item because the cellular defense against lipotoxicity provides a reasonable, yet currently partly speculative functional relationship between FA desaturation and the development of diabetes. Experimental data support that a limited cellular capacity to convert SFAs to unsaturated ones may render *β*-cells sensitive to FA-induced damages and increase the risk of *β*-cell failure [47,48]. This is because *β*-cells are particularly sensitive to SFAs [49,50], and SCD enzymes allow cells to convert these highly deleterious molecules into less harmful UFAs, thus providing an intrinsic defense mechanism against lipotoxicity [51,52]. A genetic predisposition to lower the SCD expression, therefore, might contribute to the development of diabetes mellitus, however, to the extent that this may occur is not yet clear.

In conclusion, the cell line-specific activity and FA insensitivity of the *SCD5* promoter, as well as its tissue expression pattern that is different from that of *SCD1*, together imply that there is significantly different transcriptional regulation between the two human desaturase isoforms, thereby emphasizing their likely different role in lipid metabolism. In addition, the *SCD5* promoter and its polymorphisms may represent a common denominator between T1DM and T2DM, which otherwise have mostly non-overlapping genetic backgrounds, but in a not yet fully elucidated manner. At the same time, these results raise several additional questions that may open new avenues in research to understand SCD5. The identification of protein and lipid components that regulate the tissue- and cell line-specific expression of *SCD5* may also be a new research area. Although the role of SCD1 in the development of obesity-related conditions is a relatively clear and widely investigated topic, the potential relationship of SCD5 in diabetes is only now beginning to be noticed.

## Figures and Tables

**Table 1:** Three-step transcription factor binding site screening. The position of SNPs is counted upstream from the ATG start codon.

SNP ID	Position	Allele	All Hits (above 1% Relative Score)	At Least 15% Relative Score Difference between Alleles	Relative Score Grater than 80% at Least for One Allele
rs6841081	−254	G	56,353	438	6
		T	56,356		
rs3811792	−316	C	56,359	372	9
		T	56,362		

**Table 2:** List of transcription factors that were affected by rs6841081 polymorphism. Positive values of relative score differences on a red background indicate that the minor allele increased the TF binding probability, negative values on a green background indicate that the minor allele decreased the TF binding probability. A darker shade indicates that there is a larger difference.

Name	TF ID	Strand	Relative Score (%)		
			G Allele	T Allele	Difference
SPI1	MA0080.1	-	61.12	81.85	20.73
MEIS3	MA0775.1	-	64.05	81.49	17.44
SOX18	MA1563.1	+	67.49	83.78	16.28
NFIA	MA0670.1	+	66.42	82.29	15.87
TFE3	MA0831.1	-	80.86	64.07	−16.79
TFAP2A	MA0003.1	-	86.43	67.53	−18.90

**Table 3:** List of transcription factors that were affected by rs3811792 polymorphism. Positive values of relative score differences on a red background indicate that the minor allele increased the TF binding probability, negative values on a green background indicate that the minor allele decreased the TF binding probability. A darker shade indicates that there is a larger difference.

Name	TF ID	Strand	Relative Score (%)		
			C Allele	T Allele	Difference
SOX10	MA0442.1	+	61.69	80.51	18.83
SOX2	MA0143.4	-	69.17	85.12	15.95
MEIS2	MA0774.1	+	68.63	83.88	15.25
RBPJ	MA1116.1	-	72.83	87.87	15.04
NFATC2	MA0152.1	+	91.46	75.36	−16.10
ZNF354C	MA0130.1	+	86.87	68.98	−17.89
NFATC3	MA0625.2	-	93.30	74.36	−18.94
MZF1	MA0056.1	-	81.31	61.97	−19.34
ETS1	MA0098.1	+	93.09	71.50	−21.60

**Table 4:** Comparison of genotype frequencies of rs6841081 and rs3811792 polymorphisms in control, T1DM and T2DM populations. MAF: minor allele frequency; OR: odds ratio; CI confidence interval.

		**Control** (**N = 350**)		**T1DM** (**N = 145**)		**T2DM** (**N = 253**)	
**rs6841081**		N	%	N	%	N	%
	**GG**	342	98	141	97	243	96
	**GT**	8	2	4	3	10	4
	**TT**	0	0	0	0	0	0
	***χ*2**			*p* = 0.7555		*p* = 0.1119	
	**MAF** (**T**)	8	1	4	1	10	2
	**OR with 95% CI**			1.2128 (0.3594–4.0922)		1.7593 (0.6844–4.5224)	
		**Control** (N = 350)		**T1DM** (N = 143)		**T2DM** (N = 248)	
**rs3811792**		N	%	N	%	N	%
	**CC**	248	71	97	68	162	65
	**CT**	94	27	33	23	79	32
	**TT**	8	2	13	9	7	3
	***χ*2**			***p* = 0.0029**		***p* = 0.0114**	
	**MAF** (**T**)	110	16	59	21	93	19
	**OR with 95% CI**			4.275 (1.7318–10.5527)		1.2907 (0.9108–1.8291)	

## Data Availability

Date is contained within the article.

## Supplementary Materials

The following supporting information can be downloaded at: https://www.mdpi.com/article/10.3390/genes13101784/s1, Figure S1: Effect of different fatty acids on pGL3B luciferase activity in HEK293T cells; Table S1: The list of cloning and mutagenic primers used in *SCD5* promoter and SNP analysis.
