# Supplementary material for: A Single Nucleotide Polymorphism (rs3811792) Affecting Human SCD5 Promoter Activity Is Associated with Diabetes Mellitus
Source: Genes (Basel). 2022 Oct 3;13(10):1784. doi: 10.3390/genes13101784 (PMC9601412; doi:10.3390/genes13101784)
Supplement: Supplementary file 1 [file genes-13-01784-s001.zip › genes-1925615-supplementary.pdf]

Table S1. ***The list of cloning and mutagenic primers used in SCD5 promoter and SNP analysis.*** Bold and underlined letters indicate the recognition site of the endonuclease or the nucleotide to be replaced.

	Primer name	Sequence (5' → 3')	T_A (°C)
Cloning	SCD5-P1-S-KpnI	AAA TTT **GGT ACC** ATA TGG GGG ATA CGC CAG CA	56
	SCD5-P2-S-KpnI	AAA TTT **GGT ACC** GAG CCA CCT TCC ACC CG	
	SCD5-P3-S-KpnI	AAA TTT **GGT ACC** TAA GGA GGC GTT GCA GAA GTG	
	SCD5- P4-S-KpnI	AAA TTT **GGT ACC** ACA TTT GTT TGC TCC ATC TTT GC	
	SCD5-P-AS-HindIII	AAA TTT **AAG CTT** GCA GAA AGG GAT CTT CCC CG	
Mutagenesis	rs6841081-S	TCT TTC TCG C**T**G CCG AGT TCA GCC CGG GCA GC	72
	rs6841081-AS	GGA GGC GCG CGC GGG GCG	
	rs3811792-S	CGT TCC ATT T**T**C ACA GCT CCT CCT CCC C	72
	rs3811792-AS	GGG CCG GCG ACG CTG GAG	

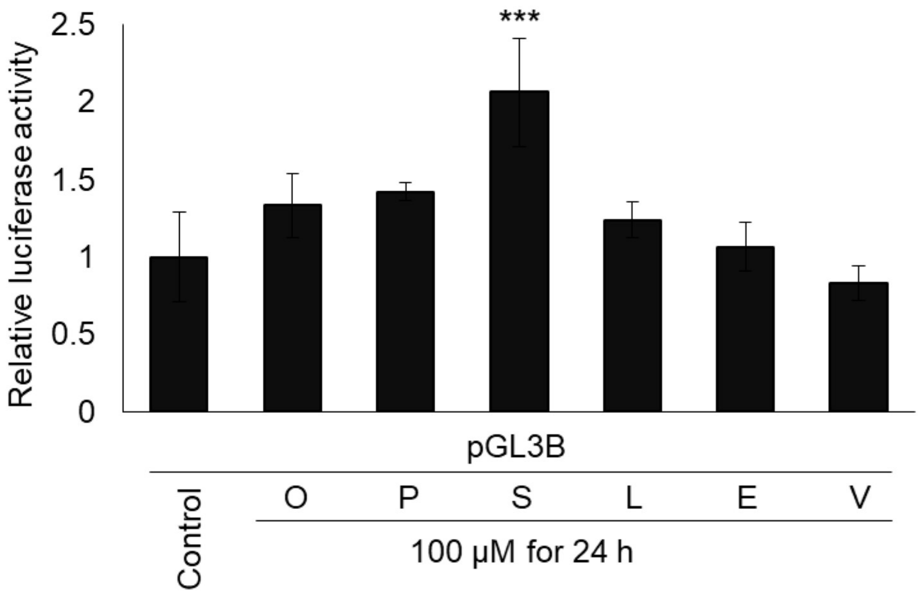

Figure S1. ***Effect of different fatty acids on pGL3B luciferase activity in HEK293T cells.*** Transfection and FA treatment were performed as described in *Materials and Methods*. pCMV-β-gal vector served as transfection control. Luciferase and β-galactosidase enzyme activities were measured as indicated in *Materials and Methods* and their relative ratios are shown as bar graphs. The diagram depicts the results of three independent measurements normalized to pGL3B promoter-less untreated vector. Data are shown as mean values ± S.D. Statistical analysis was performed by using the Tukey-Kramer Multiple Comparisons Test. Three asterisks: $p < 0.001$.
